# PRINCIPLES OF QUEER GERONTOLOGY

**DOI:** 10.1093/geroni/igae098.1944

**Published:** 2024-12-31

**Authors:** Vanessa Fabbre, Austin Oswald, Sarah Jen

**Affiliations:** Chair; Co-Chair; Discussant

## Abstract

This symposium will present principles for queer gerontology – or gerontological scholarship aimed at promoting social justice for all, but especially for LGBTQ+ people as they age – and potential applications of this approach within existing and future scholarly projects. Building upon recent applications of queer theory and perspectives to aging studies and gerontology, we propose five principles of queer gerontology—1) Deeping Visibility and Awareness; 2) Developing and Applying Critical Perspectives; 3) Challenging Oppression and Injustice; 4) Engaging Multiple Epistemologies and Modes of Inquiry; and 5) Fostering Transdisciplinary Advancement—and present evidence and themes from existing empirical and conceptual scholarship that exemplify these principles. The first presentation will describe the need for queer gerontology, the iterative process through which these principles were developed, and present the principles. The second presentation will apply a critical secondary qualitative data analysis to illustrate the principles of queer gerontology in action. The third presentation will explore the theme of being a scholar-activist in the practice of queer gerontology. The symposium will offer a chance for attendees to reflect critically on their own scholarship and devise directions for future work with respect to these principles.

